# Supplementary material for: Meta-Analysis and Gene Set Enrichment Relative to ER Status Reveal Elevated Activity of MYC and E2F in the “Basal” Breast Cancer Subgroup
Source: PLoS One. 2009 Mar 9;4(3):e4710. doi: 10.1371/journal.pone.0004710 (PMC2650420; doi:10.1371/journal.pone.0004710)
Supplement: Dataset S1 — GSEA results from first screen. The file contains a zipped archive of the HTML results of the second GSEA screen. Reports for enriched or depleted datasets may be accessed from the “index.html” link within the my_analysis.GseaPreranked.1219975950625.rpt.zip ZIP archive. Enrichment plots are available for each of the 50 most enriched and depleted gene sets. (13.32 MB ZIP) [file pone.0004710.s001.zip › my_analysis.GseaPreranked.1219975950625.rpt.zip!//VANTVEER_BREAST_OUTCOME_GOOD_VS_POOR_UP.html]

Details for gene set VANTVEER\_BREAST\_OUTCOME\_GOOD\_VS\_POOR\_UP[GSEA]

|  || Dataset | zSco.ERneg.to.ERpos\_collapsed |
| Phenotype | NoPhenotypeAvailable |
| Upregulated in class | na\_neg |
| GeneSet | VANTVEER\_BREAST\_OUTCOME\_GOOD\_VS\_POOR\_UP |
| Enrichment Score (ES) | -0.827147 |
| Normalized Enrichment Score (NES) | -2.822458 |
| Nominal p-value | 0.0 |
| FDR q-value | 0.0 |
| FWER p-Value | 0.0 |
Table: GSEA Results Summary

  

Fig 1: Enrichment plot: VANTVEER\_BREAST\_OUTCOME\_GOOD\_VS\_POOR\_UP      
 Profile of the Running ES Score & Positions of GeneSet Members on the Rank Ordered List

  

| PROBE | GENE SYMBOL | GENE\_TITLE | RANK IN GENE LIST | RANK METRIC SCORE | RUNNING ES | CORE ENRICHMENT || 1 | RBP3 |  |  | 3444 | 1.733 | -0.2390 | No |
| 2 | FGF18 |  |  | 4851 | 1.206 | -0.3308 | No |
| 3 | WISP1 |  |  | 6853 | 0.534 | -0.4751 | No |
| 4 | RPS4X |  |  | 7738 | 0.218 | -0.5391 | No |
| 5 | BBC3 |  |  | 9752 | -0.695 | -0.6824 | No |
| 6 | GCN1L1 |  |  | 10870 | -1.440 | -0.7498 | No |
| 7 | ACADS |  |  | 11005 | -1.571 | -0.7417 | No |
| 8 | KIAA1324 |  |  | 12142 | -2.762 | -0.7952 | Yes |
| 9 | QDPR |  |  | 12196 | -2.838 | -0.7664 | Yes |
| 10 | MATN3 |  |  | 12441 | -3.165 | -0.7481 | Yes |
| 11 | PEX12 |  |  | 12555 | -3.341 | -0.7180 | Yes |
| 12 | RAB27B |  |  | 12576 | -3.378 | -0.6804 | Yes |
| 13 | RAI2 |  |  | 12622 | -3.475 | -0.6436 | Yes |
| 14 | AP2B1 |  |  | 12670 | -3.557 | -0.6060 | Yes |
| 15 | TGFB3 |  |  | 12734 | -3.702 | -0.5679 | Yes |
| 16 | FBP1 |  |  | 12849 | -4.003 | -0.5302 | Yes |
| 17 | BTG2 |  |  | 13039 | -4.613 | -0.4911 | Yes |
| 18 | SEC14L2 |  |  | 13076 | -4.733 | -0.4391 | Yes |
| 19 | KIF3B |  |  | 13084 | -4.771 | -0.3844 | Yes |
| 20 | PIB5PA |  |  | 13125 | -4.921 | -0.3305 | Yes |
| 21 | FUT8 |  |  | 13195 | -5.349 | -0.2738 | Yes |
| 22 | PECI |  |  | 13201 | -5.385 | -0.2119 | Yes |
| 23 | CIRBP |  |  | 13206 | -5.436 | -0.1494 | Yes |
| 24 | GSTM3 |  |  | 13252 | -5.994 | -0.0834 | Yes |
| 25 | KRT18 |  |  | 13317 | -7.649 | 0.0002 | Yes |
Table: GSEA details [plain text format]

  

Fig 2: VANTVEER\_BREAST\_OUTCOME\_GOOD\_VS\_POOR\_UP: Random ES distribution      
 Gene set null distribution of ES for **VANTVEER\_BREAST\_OUTCOME\_GOOD\_VS\_POOR\_UP**
